# What is the safe noise exposure level to prevent noise-induced hearing loss?

**DOI:** 10.1038/s41370-024-00660-3

**Published:** 2024-04-18

**Authors:** Daniel Fink

**Affiliations:** The Quiet Coalition, Concord, MA USA

**Keywords:** Health Studies, Personal Exposure, Population Based Studies

## Introduction

Exposure to noise causes noise-induced hearing loss (NIHL) [1] and two other auditory disorders, tinnitus and hyperacusis [2]. This Comment will focus on answering the question, “What is the safe noise exposure level to prevent NIHL?” The exposure-response relationship between noise and hearing loss in humans has been studied in the occupational setting for decades [3]. Based on thousands of laboratory studies in a variety of animal models, the mechanisms by which noise exposure causes NIHL are also well understood, down to the ultrastructural, biochemical, and genetic effects of noise on cochlear hair cells and synaptic junctions [4, 5]. The exposure-response relationships for tinnitus and hyperacusis have not been established, though, and the mechanisms of injury are not yet understood. Ninety per cent of people with tinnitus also have hearing loss [6]. Knowledge of the safe noise exposure level to prevent NIHL should also help people avoid developing noise-induced tinnitus, and probably hyperacusis as well.

## Noise causes hearing loss

It has been known since the eighteenth century, if not earlier, that men working in certain occupations- blacksmiths, stonemasons, and bell ringers among them- couldn’t hear well. After the development of gunpowder, hearing loss became common in soldiers and sailors [7]. The first report of occupational noise-induced hearing loss (NIHL) is said to be that of Ramazzini in 1713 among coppersmiths in Venice [8]. During the industrial age, hearing loss in workers making steam boilers was so common that it became known as boilermaker’s disease [7]. The U.S. National Institute for Occupational Safety and Health (NIOSH) was established in 1970, and published recommended exposure limits for occupational noise in 1972 [9]. These recommendations were updated in 1998. NIOSH is part of the U.S. Centers for Disease Control and Prevention (CDC) but it wasn’t until 2015 that CDC recognized that noise exposure caused NIHL in the public, not just in workers with occupational exposure [10].

**Fig. 1:**
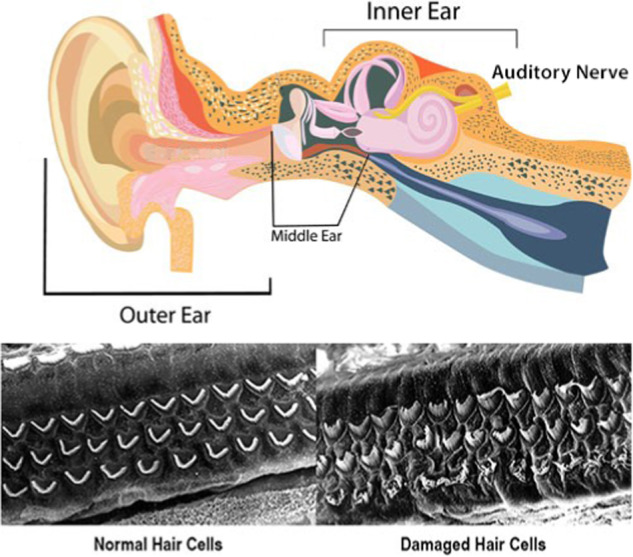
Top: Auditory structures from external ear (pinna) to auditory nerve. Bottom: Normal and damaged hair cells. From Centers for Disease Control and Prevention. How does loud noise cause hearing loss? [11].

The anatomy of the auditory system is illustrated in Fig. 1 [11]. The physiology of hearing and the details of mechanotransduction are well described [12]. Sound waves collected and focused by the external ear (pinna) cause vibrations in the ear drum (tympanic membrane) which are communicated via three tiny bones in the middle ear to the cochlea, where they cause distortion of cochlear hair cells, the basic sensory organ of hearing. The hair cell distortions in turn cause chemical changes transduced into electrical impulses, which are transmitted via cochlear synapses to the auditory nerve, and thence to the auditory processing cortex in the brain where they are perceived as sound.

The mechanisms by which loud noise damages cochlear structures are well-understood, down to the ultrastructural, molecular, and genetic levels [4, 5]. The damage noise exposure does to cochlear hair cells, the basic sensory receptors for hearing, is shown at the bottom in Fig. 1. Animal research over the last two decades has also demonstrated that noise damages cochlear synapses [13], with recent confirmation of the same effects in post-mortem studies of human temporal bone specimens [14]. This damage is thought to be the major cause of speech-in-noise difficulty, the difficulty following one conversation among many in a noisy environment. Speech-in-noise difficulty is called hidden hearing loss because patients complaining of difficulties understanding speech often have normal or near normal audiograms. The prevalence of speech-in-noise difficulty is reported to be 10–15% of the adult population, but since speech-in-noise testing is not done during screening audiometry, it may be higher [15].

The Equal Energy Hypothesis states that equal amounts of sound energy will produce equal amounts of hearing impairment, regardless of how the sound energy is distributed in time [9]. A useful albeit imperfect analogy for the effect of noise exposure on the ear is the effect of sun exposure on the skin. Both NIHL and deep wrinkles and pigment changes are the results of exposures to energy, the first of cochlear hair cells to sound energy and the second of the skin to solar energy. Drooping of the skin (ptosis) is part of normal aging, due to the downward force of gravity on collagen fibers, but without sun exposure, the skin remains smooth and unwrinkled into old age [16]. Without excessive noise exposure, auditory sensitivity (hearing) remains normal into old age [17]. The analogy is imperfect because ultraviolet components of sunlight cause direct DNA damage in the skin, whereas noise exposure can damage inner ear structures directly and also leads to chemical changes damaging or killing cochlear hair cells.

Average noise exposure measurements obscure the impact of brief high-intensity noise exposures, called impulse or impulsive noise, which have a disproportionate impact on auditory health [18, 19]. Intermittent noise exposure is difficult to study in the occupational setting, and is subsumed into calculated recommended averages for occupational noise exposure [9], but this may underestimate the impact of non-Gaussian noise exposure [18]. The effect of impulse noise on the public has not been systematically studied, with only anecdotal news and case reports of impulse noise exposure causing hearing loss, tinnitus, or hyperacusis. For both occupational and non-occupational noise exposure, greater attention must be paid to impulse noise. A dermatologic analogy for the disproportionate impact of impulsive noise on hearing may be the fact that one severe sunburn in childhood or adolescence has been correlated with the development of melanoma in adult years [20].

## Noise induced hearing loss is a major cause of disability

NIHL is a major problem in the United States and the world. Approximately 25% of American adults age 20–69 have noise-induced hearing loss, half with no significant occupational noise exposure [21]. According to the CDC, hearing loss is the third most common chronic physical condition in the United States [22]. Globally, an estimated 5% of the world’s population has NIHL [1]. The 2019 Global Burden of Disease Study found that hearing loss is the fourth leading cause of disability globally [23]. In the United States and Europe, approximately 30–50% of adults over age 65 have hearing loss great enough to affect communication [24, 25]. The prevalence of hearing loss increases to approximately 80% over age 80, with almost everyone reaching the tenth decade of life having hearing loss [26].

There are many causes of hearing loss- infections, ototoxic drugs, genetic diseases among them- but the most common cause of hearing loss with age is NIHL, the result of a lifetime of cumulative excess noise exposure [17, 27]. Hearing loss is not a benign condition. In addition to communication difficulties, which in younger individuals can affect success in school and in the workplace leading to reduced lifetime earnings [28], hearing loss in older people is correlated with many adverse health effects. These include increased risk of falls, social isolation, depression, dementia, accidents, and hospitalization and death [29]. The only current approved treatments for hearing loss are amplification (with hearing aids) and cochlear implantation, the latter reserved for the profoundly hearing impaired [29]. There is a stigma associated with hearing loss [30] and a high non-usage rate for those who have acquired hearing aids [31]. Unfortunately, hearing aids do not restore normal hearing and do not provide an auditory correction similar to the visual correction provided by lenses [32]. Hearing aids are also costly, and no country can afford to provide them to all its citizens who need them. But even in countries where hearing aids are provided by national health programs, there are still many people who do not wear hearing aids [33]. Hearing aid non-use may be common because while hearing aids help people hear better in quiet ambient noise situations, amplification is less helpful in high ambient noise situations [34]. Newer digital hearing aids with tunability and frequency band adjustment features are advertised as being more helpful than older analog models, but as yet no published peer-reviewed research has confirmed this. Perhaps more importantly, it is obvious from looking at the photomicrographs in Fig. 1 that delivering amplified sound waves to dead or damaged cochlear hair cells is unlikely to help hearing as much as desired.

## Hearing loss is not part of normal aging

**Fig. 2:**
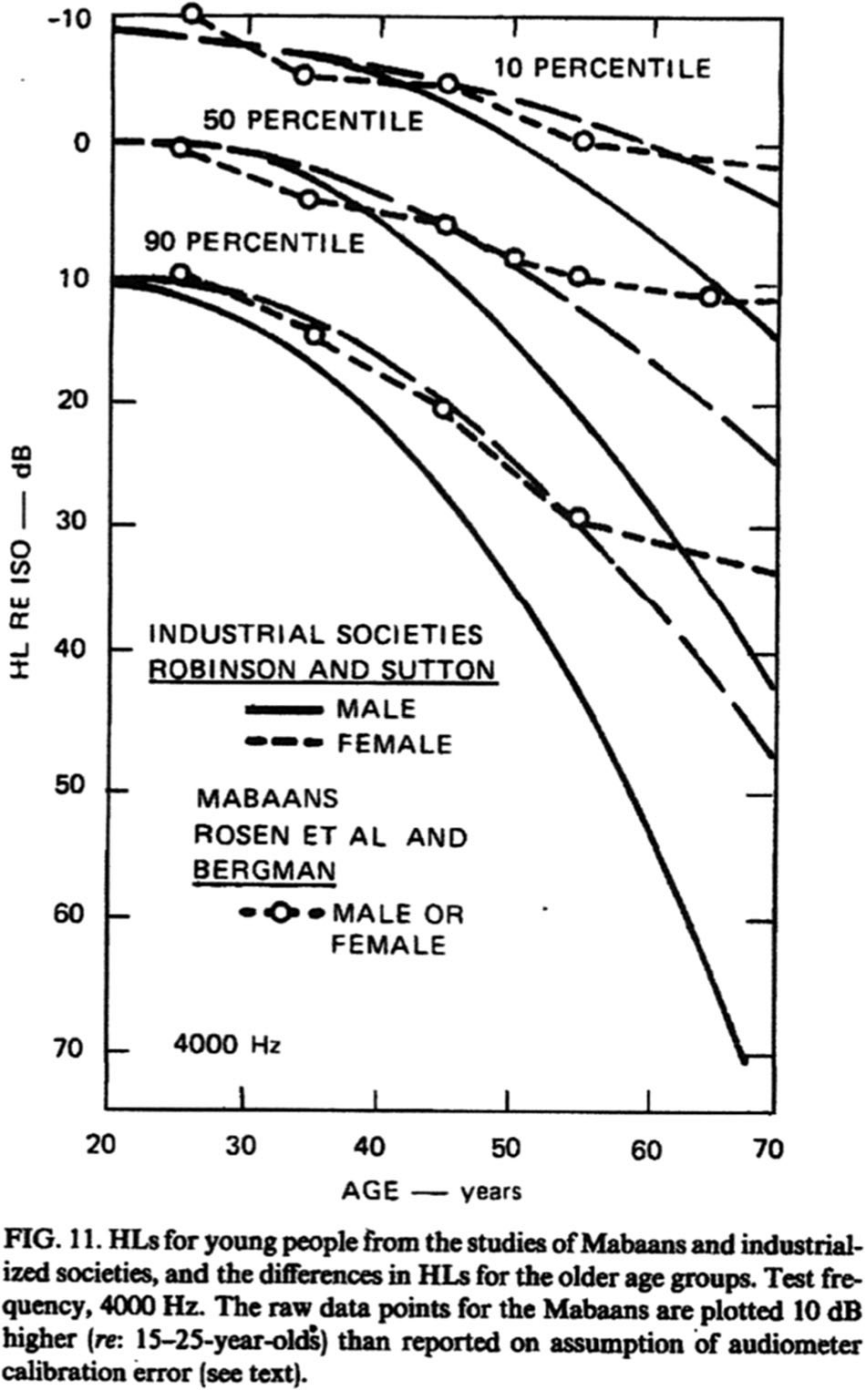
Hearing levels for Mabaans and industrialized societies. Figure is reproduced with permission of the Acoustical Society of America from ref. [17].

What is often called *age-related hearing loss* or *presbycusis* largely represents the effects of cumulative lifetime noise exposure on the ears [27]. Figure 2 shows that actual *age-related hearing loss* in a population not exposed to loud noise is approximately only a 10 dB decrement at age 70 [17]. This hearing threshold level does not meet standard criteria for hearing loss [35].

It has been postulated that factors other than noise exposure are important causes of hearing loss with age, e.g., genetic factors, exposure to ototoxic substances, diabetes, smoking, hypertension, or atherosclerosis. These factors and others are indeed correlated with hearing loss. However, studies done in the 1960s in isolated populations not exposed to loud noise found preservation of auditory sensitivity into old age. The best known of these may be that by Rosen et al. in the Mabaan population of the Sudan [36].

The importance of noise exposure as a cause of hearing loss was subsequently demonstrated in the 1986 study by Goycoolea et al. [37]. Using a natural experiment study design, they found that hearing loss was more prevalent in Easter Island residents who had left the remote, very quiet island to seek employment on the noisier South American mainland than in those who had remained at home. They concluded that noise exposure the most important factor, stating that,

*“With all factors being equal, except exposure to modern civilization, our results showed that living in civilized societies has a significant negative effect on hearing; the severity is directly proportional to the years of exposure.”*

The fact that noise was the most important contributor to hearing loss in old age was confirmed by the 2020 study of donated temporal bone specimens by Wu et al. [14]. They stated,

*“…the larger, and more functionally significant, basal loss in humans is largely noise-induced. If true, the bad news is that we are all abusing our ears, to our significant functional detriment, as we age.”*

Most people living in industrialized societies are exposed to everyday noise levels sufficient to cause NIHL [38, 39]. but are almost entirely unaware that they are “abusing their ears.”

## Preventing noise-induced hearing loss

Prevention of disease is better and less expensive than treatment or rehabilitation [40]. The U.S. Centers for Disease Control and Prevention state that “hearing loss from noise is 100% preventable” [41]. Again, what is the actual safe noise exposure level to prevent noise-induced hearing loss (NIHL)? This cannot be the NIOSH recommended exposure limit (REL) of 85 A-weighted decibel (dbA) for occupational noise, first calculated in 1972 and revised in 1998 [9]. Occupational noise exposure limits do not prevent NIHL, even if they are often wrongly cited as safe for the public or as the sound pressure level at which auditory damage begins [42]. The NIOSH REL allows an 8% excess risk of occupational NIHL; the 90 dBA U.S. Occupational Safety and Health Administration permissible exposure limit allows a 25% excess risk [9]. Even if members of the public are not exposed to noise 8 h/day, 50 weeks/year, for 40 years, these are not safe noise exposure levels, not for workers, certainly not for the public, and especially not for children.

The only evidence-based safe noise exposure level to prevent NIHL, the U.S. Environmental Protection Agency’s (EPA) calculated 70 dB time-weighted daily average (Leq_(24)_ = 70) for the public [43, 44], can no longer be considered safe, either. One reason the EPA’s 70 dB level may not prevent NIHL is that, as discussed above, disproportionate auditory damage can be caused by brief high-intensity noise exposures obscured by average noise exposure measurements, recommendations, or calculations. More importantly, everyday noise exposure now begins in early childhood and continues at home and from recreational activities during working years and then after retirement.

Consequently, both occupational noise exposure limits and the EPA’s safe noise level must be revised downwards to reflect increased non-occupational noise exposure. For both occupational exposure limits and public noise exposure calculations, three additional factors must be considered [45]: 1) cumulative lifetime noise exposure, now approaching 80 years, not just 40-year adult noise exposure histories; 2) detection of noise-induced auditory damage by more sensitive methods than limited-frequency pure tone audiometry, such as extended range audiometry, speech-in-noise testing, and questions about tinnitus and hyperacusis [46]; and 3) use of a zero hearing threshold level rather than 15 dB hearing threshold level used by NIOSH as the standard for normal hearing [47].

## What is the actual safe noise exposure level to prevent nihl?

Why does knowing the actual safe noise exposure level matter? Without knowing the safe noise exposure level, it is impossible to accurately advise both workers and the public on how to protect their hearing. For the public, if a condition is an inevitable part of normal physiological aging, e.g., thinning, graying hair, nothing can be done to prevent it. If the condition is not inevitable, e.g., muscle weakness, obesity, hypertension, and diabetes, behavioral changes can prevent or at least delay the onset of the condition [27]. For NIHL, avoidance of loud noise exposure or use of hearing protection devices can prevent the development of what is commonly called age-related hearing loss.

**Fig. 3:**
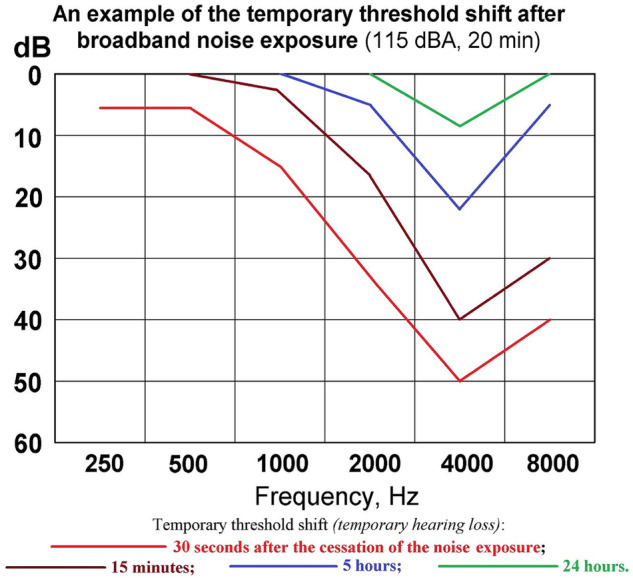
Noise-induced temporary threshold shift. From ref. [56]. Published under Creative Commons CO 1.0 Universal Public Doman Dedication.

How can we answer the question, “What is the actual safe noise exposure level to prevent NIHL?” Due to modern ethical and legal protections for human research subjects, one cannot design a study purposefully exposing them to sufficient noise to damage their hearing to assess how much noise exposure causes hearing loss. A > 80-year observational study correlating measured or estimated lifetime noise exposure with hearing loss would be costly and difficult to complete. Fortunately, historical studies may provide an answer. Before modern research subject protections were established, noise-induced temporary threshold shift (NITTS), the temporary decrease in auditory sensitivity after loud noise exposure, was used as a measure of auditory damage from noise [48]. NITTS is seen immediately after noise exposure, but largely resolves over time (See Fig. 3.).

With repeated exposures, NITTS eventually becomes noise-induced permanent threshold shift, i.e., NIHL. This persistent damage is suggested by the residual decrease in auditory sensitivity at 24 h after exposure in the green line in Fig. 3. Figure 3 also shows the audiometric notch, the concentration of hearing loss around 4 kHz, which is considered pathognomonic for NIHL [49].

NITTS is a real and measurable phenomenon. Additionally, any auditory symptoms after noise exposure, including tinnitus, likely indicate that permanent auditory damage has occurred [50]. The sound pressure level needed for the human ear to recover from NITTS, the effective quiet level, is approximately 55 dBA [51]. This is probably the safe noise exposure level to prevent NIHL from a single exposure, with 55–60 dB time-weighted average being the actual safe noise exposure level for a day.

This analysis is not new. Almost 30 years ago, Kryter wrote,

*“Several investigators, using human and animal subjects, have found that recovery from Temporary Threshold Shift is reduced when the level of background noise in periods between exposures to more intense noise was no higher than L*_*A*_ *50–70* *dB. Not until the “noise” in the recovery periods was less than those levels did full recovery continue. This maximum level, perhaps for humans around L*_*A*_*55 for an octave band and L*_*A*_*60 for broadband noise, is called effective quiet, and presumably indicates a level, perhaps a 24-h, EL*_*Aeq,24h*_*, energy level, required for complete avoidance by the average, and 50%ile, ear of sound induced permanent threshold shifts during a lifetime* [51].

The 55 dBA effective quiet level likely represents the sound pressure level at which reversible intracellular chemical processes involved in hearing are overwhelmed, eventually causing noise-induced hearing loss. This hypothesis must be confirmed by animal studies. Fifty-five dBA is approximately the sound pressure level of human speech in a quiet environment [52]. It appears that humans evolved to be able to communicate with each other without damaging our hearing, but any sounds greater than the relatively low sound pressure level of speech may cause auditory damage.

## Conclusion

Based on Kryter’s analysis, the safe noise exposure level to prevent NIHL is about 55–60 dB time-weighted average for a day. Since any temporary auditory discomfort, tinnitus, or NITTS likely indicates that permanent auditory damage has occurred, it is possible that the safe noise exposure level for impulse noise is also only 55 dBA. These sound pressure levels are radically lower than current occupational noise exposure recommendations, the EPA’s calculated safe noise level, or any published guidance for public noise exposure. There is no reason to question Kryter’s 30-year old analysis of human data, but these proposed safe noise levels need to be confirmed by laboratory studies using appropriate animal models.

Terminology matters. The commonly used terms *presbycusis* and *age-related hearing loss* misleadingly imply that hearing loss is an inevitable part of normal aging. More accurate terms may be Kryter’s *sociocusis* [17] or *noise-induced hearing loss in the elderly* (*NIHL-E*). The standard definition of noise, *noise is unwanted sound*, does not accurately reflect the harm that noise does. Wanted sound, e.g., at a rock concert or from power tool use, can cause NIHL, and unwanted sound is stressful and has adverse non-auditory health effects. A better definition is *noise is unwanted and/or harmful sound* [53]. This new definition of noise opens the abstract of the 2021 American Public Health Association policy statement *Noise as a Public Health Hazard* [54] and was adopted for use in 2023 by the International Commission on Biological Effects of Noise and added to its Constitution [55].

Three things- the new definition of noise, an understanding that hearing loss with age is not part of normal physiological aging but largely represents noise damage, and public awareness of a lower safe noise exposure level to prevent NIHL- may prompt at least some individuals to reduce noise exposure for themselves and their children. Lower occupational noise exposure limits may lead to recommendations or regulations for lower public noise exposure. Even if wrongly cited as safe for the public, lower occupational noise limits would also reduce public noise exposure. CDC states that NIHL is the only type of hearing loss that is entirely preventable. Prevention of disease is better and less expensive than treatment. Knowledge that both wanted and unwanted noise are harmful, combined with awareness of the actual safe noise exposure level to prevent NIHL, may help both workers and members of the public prevent NIHL.

## References

[1] Natarajan N, Batts S, Stankovic KM. Noise-induced hearing loss. J Clin Med. 2023;12:2347. 36983347 10.3390/jcm12062347 PMC10059082

[2] Zeng FG. Tinnitus and hyperacusis: central noise, gain and variance. Curr Opin Physiol. 2020;18:123–9. 33299958 10.1016/j.cophys.2020.10.009 PMC7720792

[3] Themann CL, Tisdale-Pardi J, Kardous CA, Masterson EA, Morata TC, Murphy WJ. NIOSH noise: a 50-year timeline of research and intervention. NIOSH Science Blog. 2022. https://stacks.cdc.gov/view/cdc/119462.

[4] Kurabi A, Keithley EM, Housely GD, Ryan AF, Wong AC-Y. Cellular mechanisms of noise-induced hearing loss. Hear Res. 2017;349:1290137. 10.1016/j.heares.2016.11.013 PMC6750278 27916698

[5] Wagner EL, Shin J-B. Mechanisms of hair cell damage and repair. Trends Neurosci. 2019;42:414–24. 30992136 10.1016/j.tins.2019.03.006 PMC6556399

[6] Dobie RA. Overview: suffering from tinnitus. In Snow JB, editor. Tinnitus: Theory and Management. New Haven, CT: PMPH-USA; 2004.

[7] Owen D. Volume Control: Hearing in a Deafening World. New York: Riverhead Books; 2019.

[8] Murphy WJ. Preventing occupational hearing loss- time for a paradigm shift. Acoust Today. 2016;12:28–35.

[9] National Institute for Occupational Safety and Health. Criteria for a Recommended Standard: Occupational Noise Exposure. Cincinnati (OH): National Institute for Occupational Safety and Health; 1998. https://stacks.cdc.gov/view/cdc/6376.

[10] Eichwald J, Benet L. CDC addresses non-occupational noise-induced hearing loss. Hear J. 2020;73:16–17. 10.1097/01.hj.0000719788.78887.02 PMC9348587 35928763

[11] Centers for Disease Control and Prevention. How does loud noise cause hearing loss? Updated November 24, 2020. https://www.cdc.gov/nceh/hearing_loss/how_does_loud_noise_cause_hearing_loss.html Accessed January 27, 2024.

[12] Stover T, Diensthuber M. Molecular biology of hearing. GMS Curr Top Otorhinolaryngol Head Neck Surg. 2011;10:Doc06. 22558056 10.3205/cto000079 PMC3341583

[13] Fernandez KA, Guo D, Micucci S, De Gruttola V, Liberman MC, Kujawa SG. Noise-induced cochlear synaptopathy with and without sensory cell loss. Neuroscience. 2020;427:43–57. 31887361 10.1016/j.neuroscience.2019.11.051 PMC7450393

[14] Wu P, O’Malley JT, de Gruttola V, Liberman MC. Age-related hearing loss is dominated by damage to inner ear sensory cells, not the cellular battery that powers them. J Neurosci 2020;40:6457–6366. 10.1523/JNEUROSCI.0937-20.2020 PMC7424870 32690619

[15] Kohrman DC, Wan G, Cassinotti L, Corfas G. Hidden hearing loss: a disorder with multiple etiologies and mechanisms. Cold Spring Harb Perspect Med. 2020;10:a035493. 30617057 10.1101/cshperspect.a035493 PMC6612463

[16] Flament F, Bazin R, Laquieze S, Rubert V, Simonpietri E, Piot B. Effect of the sun on visible clinical signs of aging in Caucasian skin. Clin Cosmet Investig Dermatol. 2013;27:221–32. 10.2147/CCID.S44686 PMC3790843 24101874

[17] Kryter KD. Presbycusis, sociocusis, and nosocusis. J Acoust Soc Am. 1983;73:1897–917. 10.1121/1.389580. 10.1121/1.389601 6655136

[18] Suter AH. Occupational hearing loss from non-Gaussian noise. Semin Hear. 2017;38:225–62. 28740322 10.1055/s-0037-1603726 PMC5520237

[19] Mantyselo S, Vuori J. Effects of impulse noise and continuous steady state noise on hearing. Br J Ind Med. 1984;41:122–32. 6691929 10.1136/oem.41.1.122 PMC1009246

[20] Wu S, Cho E, Li W-Q, Weinstock MA, Qureshi AA. History of severe sunburn and risk of skin cancer among women and men in 2 prospective studies. Am J Epidemiol. 2016;183:824–33. 27045074 10.1093/aje/kwv282 PMC4851991

[21] Carroll YI, Eichwald J, Scinicariello F, Hoffman HJ, Deitchman S, Radke MS, et al. Vital signs: Noise-induced hearing loss among adults- United States 2011-2012. MMWR Morb Mortal Wkly Rept 2017;666:139–44. 10.15585/mmwr.mm6605e3 PMC5657963 28182600

[22] Centers for Disease Control and Prevention. Noise and occupational hearing loss. 2023. https://www.cdc.gov/niosh/topics/noise/about.html# Accessed February 13, 2024.

[23] GBD 2019 Hearing Loss Collaborators. Hearing loss prevalence and years lived with disability, 1990-2019: findings from the Global Burden of Disease Stuey 2019. Lancet. 2021;397:996–1009. 33714390 10.1016/S0140-6736(21)00516-X PMC7960691

[24] Lin FR, Niparko JK, Ferrucci L. Hearing loss prevalence in the United States. Arch Int Med 2011;171:1851–2. 22083573 10.1001/archinternmed.2011.506 PMC3564588

[25] Roth TN, Hanebluth D, Probst R. Prevalence of age-related hearing loss in Europe: a review. Eur Arch Otorhinolaryngol. 2011;268:1101–7. 21499871 10.1007/s00405-011-1597-8 PMC3132411

[26] Reed NS, Garcia-Morales EE, Myers C, Huang AR, Ehrlich JR, Killeen OJ, et al. Prevalence of hearing loss and hearing aid use among US Medicare beneficiaries age 71 and older. JAMA Netw Open. 2023;6:E22325320. 10.1001/jamanetworkopen.2023.26320 PMC10383002 37505496

[27] Fink D. Significant hearing loss is probably not part of normal aging. Presented at the 12^th^ Congress of the International Commission on Biological Effects of Noise, Zurich, Switzerland, June 20, 2017. http://www.icben.org/2017/ICBEN%202017%20Papers/SubjectArea01_Fink_0102_2331.pdf.

[28] Kochkin S. The impact of untreated hearing loss on household income. 2007. https://betterhearing.org/HIA/assets/File/public/marketrak/MarkeTrak_VII_The_Impact_of_Untreated_Hearing_Loss_on_Household_Income.pdf.

[29] Cunningham LL, Tucci DL. Hearing loss in adults. N Engl J Med. 2017;377:2465–73. 29262274 10.1056/NEJMra1616601 PMC6457651

[30] Wallhagen MI. The stigma of hearing loss. Gerontologist. 2010;50:55–75. 10.1093/geront/gnp107 PMC2904535 19592638

[31] McCormack A, Fortnum H. Why do people fitted with hearing aids not wear them? Int J Audio. 2013;52:360–8. 10.3109/14992027.2013.769066 PMC3665209 23473329

[32] Eberts S. 10 reasons hearing aids are NOT like glasses. Huffpost. 2016. https://www.huffpost.com/entry/hearing-aids-are-not-like-glasses_b_11227882.

[33] Sternasty K, Dhar S. Barriers to hearing aid adoption run deeper than the price tag. JAMA Otolaryngol Head Neck Surg. 2021;147:498–9. 10.1001/jamaoto.2021.0172. 33764375 10.1001/jamaoto.2021.0172

[34] Fink D. Disability rights aspects of ambient noise for people with auditory disorders under the Americans with Disabilities Act. Proc Mtgs Acoust. 2017;31:015001 10.1121/2.0000657.

[35] Olusanya BO, Davis AC, Hoffman HJ. Hearing loss grades and the International Classification of functioning, disability, and health. Bull World Health Organ. 2019;97:725–8. 31656340 10.2471/BLT.19.230367 PMC6796665

[36] Rosen S, Bergman M, Plester D, El-Mofty A, Satti MH. Presbycusis study of a relatively noise-free population in the Sudan. Ann Otol Rhinol Laryngol. 1962;71:727–43. 13974856 10.1177/000348946207100313

[37] Goycoolea MV, Goycoolea HG, Farfan CR, Rodriguez LG, Martinez GC, Vidal R. Effect of life in industrialized societies on hearing in natives of Easter Island. Laryngoscope. 1986;96:1391–6. 3784745 10.1288/00005537-198612000-00015

[38] Fink D. Too loud! Non-occupational noise exposure causes hearing loss. Proc Mtgs Acoust. 2021;43:040002 10.1121/2.0001436.

[39] Pienkowski M. Loud music and leisure noise is a common cause of chronic hearing loss, tinnitus, and hyperacusis. Int J Environ Res Public Health. 2021;18:4236. 33923580 10.3390/ijerph18084236 PMC8073416

[40] Hacker K, Briss PA. An ounce of prevention is still worth a pound of cure, especially in the time of COVID-19. Prev Chronic Dis. 2021;18:200627 10.5888/pcd18.200627. 10.5888/pcd18.200627 PMC7845547 33411667

[41] Centers for Disease Control and Prevention. Understand noise exposure. 2023. https://www.cdc.gov/niosh/topics/noise/preventoccunoise/understand.html#:~:text=Noiselevelsarelikelyhazardous,bycreatingaquieterworkplace.

[42] Gerhart M. Decibel levels and hearing health: what to know. Forbes Health. 2023. https://www.forbes.com/health/hearing-aids/decibel-levels/.

[43] Environmental Protection Agency. Information on Levels of Environmental Noise Requisite to Protect Public Health and Welfare With an Adequate Margin of Safety. Washington, D.C.: EPA; 1974. https://www.nonoise.org/library/levels74/levels74.htm.

[44] Fink DJ. What is a safe noise level for the public? Am J Public Health. 2017;107:44–45. 10.2105/AJPH.2016.303527. 27925831 10.2105/AJPH.2016.303527 PMC5308171

[45] Fink D. The recommended exposure limit for occu pational noise needs to be revised downwards. Proc Mtgs Acoust. 2023;50:040002.

[46] Liberman MC, Epstein MJ, Cleveland SS, Wang H, Maison SF. Towards a differential diagnosis of hidden hearing loss in humans. PLOS One. 2016. 10.1371/journal.pone.0162726. 10.1371/journal.pone.0162726 PMC5019483 27618300

[47] Pienkowski M. On the etiology of listening difficulties in noise despite clinically normal audiograms. Ear Hearing. 2017;38:135–48. 10.1097/AUD.0000000000000388. 28002080 10.1097/AUD.0000000000000388 PMC5325255

[48] Ryan AF, Kujawa SG, Hammill T, LePrell C, Kil J. Temporary and permanent noise-induced threshold shifts: a review of basic and clinical observations. Otol Neurol. 2016;37:e271–e275. 10.1097/MAO.0000000000001071 PMC4988324 27518135

[49] McBride DI, Williams S. Audiometric notch as a sign of noise induced hearing loss. Occup Environ Med. 2001;58:46–51. 11119634 10.1136/oem.58.1.46 PMC1740031

[50] Kujawa SG, Liberman MC. Adding insult to injury: cochlear nerve degeneration after “temporary” noise-induced hearing loss. J Neursci. 2009;29:14077–85. 10.1523/JNEUROSCI.2845-09.2009 PMC2812055 19906956

[51] Kryter KD. Chapter 5. Derivation of a general theory and procedure for predicting hearing loss from sound. In: The Handbook of Hearing and the Effects of Noise: Physiology, Psychology, and Public Health. San Diego, California: Academic Press; 1994, pp. 266–268.

[52] Pearsons KL, Bennett RL, Fidell S. Speech levels in various noise environments. Report No. EPA-600/1-77-025. US Environmental Protection Agency; 1977.

[53] Fink D. A new definition of noise: noise is unwanted and/or harmful sound. Noise is the new ‘secondhand smoke. Proc Mtgs Acoust. 2019;39;050002. 10.1121/2.0001186.

[54] American Public Health Association. Policy Number 2021115. Noise as a Public Health Hazard. 2021. https://apha.org/Policies-and-Advocacy/Public-Health-Policy-Statements/Policy-Database/2022/01/07/Noise-as-a-Public-Health-Hazard#.

[55] International Commission on Biological Effects of Noise. About ICBEN. Last revision: October 2023. 2023. https://www.icben.org/About.html.

[56] Chirkin A. Temporary threshold shift (hearing loss) after noise exposure. Wikimedia Commons. Published under Creative Commons CO 1.0 Universal Public Doman Dedication. May 4, 2016. https://commons.wikimedia.org/wiki/File:Temporary_threshold_shift_(hearing_loss)_after_noise_exposure.jpg.

